# The Ilizarov method for the treatment of complex tibial fractures and non-unions in a mass casualty setting: the 2005 earthquake in Pakistan

**DOI:** 10.1007/s11751-015-0213-7

**Published:** 2015-03-13

**Authors:** Konstantinos Tilkeridis, Basavraj Chari, Nusrat Cheema, Marios Tryfonidis, Arshad Khaleel

**Affiliations:** 1 Rowley Bristow Orthopaedic Unit, St. Peter’s Hospital, Guildford Road, Chertsey, Surrey KT16 0PZ UK; 2 Trauma and Orthopaedics, Rowley Bristow Orthopaedic Unit, St. Peter’s Hospital, Guildford Road, Chertsey, Surrey KT16 0PZ UK; 3 Trauma and Orthopaedics, Northern General Hospital, Sheffield, UK; 4 University Hospital of Alexandroupolis, 6th km Alexandroupolis-Dragana, 68100 Alexandroupolis, Greece

**Keywords:** Ilizarov external fixator, Mass casualty setting, Earthquake, Complex tibial fracture, Non-union, Humanitarian mission

## Abstract

We report our experience in treating victims of the recent earthquake disaster in Pakistan. Our experience was based on two humanitarian missions to Islamabad: one in October 2005, 10 days after the earthquake, and the second in January 2006. The mission consisted of a team of orthopaedic surgeons and a second team of plastic surgeons. The orthopaedic team bought all the equipment for application of Ilizarov external fixators. We treated patients who had already received basic treatment in the region of the disaster and subsequently had been evacuated to Islamabad. During the first visit, we treated 12 injured limbs in 11 patients. Four of these patients were children. All cases consisted of complex multifragmentary fractures associated with severe crush injuries. All fractures involved the tibia, which were treated with Ilizarov external fixators. Nine fractures were type 3b open injuries. Eight were infected requiring debridement of infected bone and acute shortening. During a second visit, we reviewed all patients treated during our first mission. In addition, we treated 13 new patients with complex non-unions. Eight of these patients were deemed to be infected. All patients had previous treatment with monolateral fixators as well as soft tissue coverage procedures, except one patient who had had an IEF applied by another team. All these patients had revision surgery with circular frames. All patients from both groups were allowed to fully weight-bear post-operatively, after a short period of elevation to allow the flaps to take. Overall, all fractures united except one case who eventually had an amputation. Four patients had a corticotomy and lengthening, and three of them had a successful restoration of limb length. The fourth patient was the one with the eventual amputation.

## Introduction

In 2005, an earthquake in Pakistan caused 75,000 deaths, 150,000 injured and displaced 2.5 million people. We report our experience in applying Ilizarov’s principles [1, 2] during this disaster for patients with limb fractures associated with significant soft tissue injuries and for complex non-unions. The aim is to highlight the experience and the lessons learnt in limb salvage in a mass civilian casualty setting.

## Materials and methods

### Preparation

The team of volunteers from the UK consisted of three surgeons and two consultant anaesthetists; amongst the surgeons were a consultant, a post-fellowship Limb Reconstruction Fellow and an orthopaedic surgery trainee. A plastic surgeon was unable to accompany the team, but local arrangements were made for plastic surgeons there to assist the team. Contacts were made through three formal channels; The UK Disaster Relief Agency, The Pakistan Disaster Relief Agency and the Red Cross. Local companies were contacted for donations of orthopaedic fixation devices. Fund raising was arranged locally through mosques, schools and the media. The funds were requested to purchase the necessary equipment for the task. No funds were received by members of the team.

**Fig. 1:**
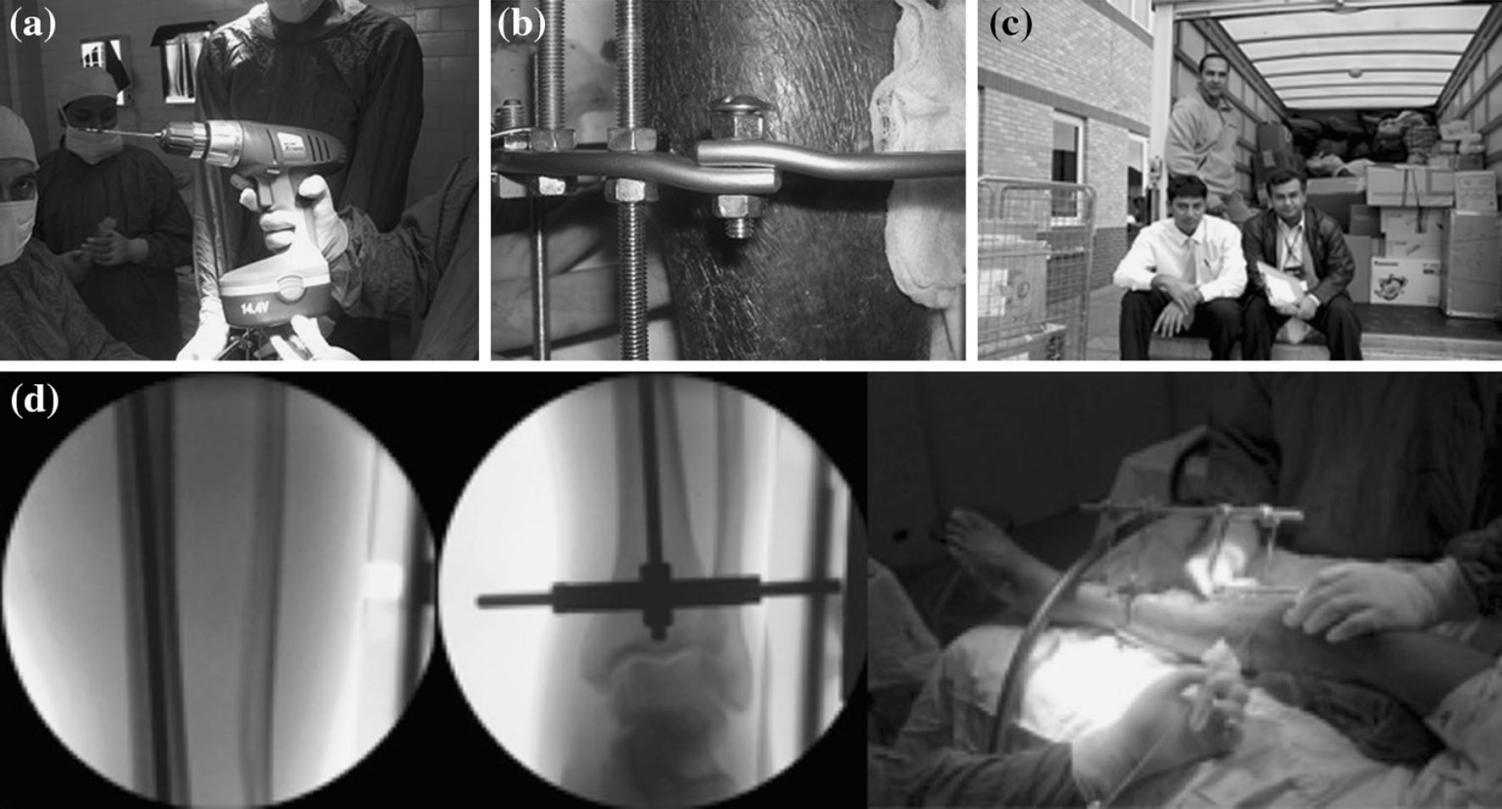
Power drivers, threaded steel rods, bolts and nuts were purchased from a hardware shop. **a** Power driver, **b** threaded steel rods, bolts and nuts, **c** the equipment and team, **d** “Sputnik” to aid reference wire placement

It was envisaged that a base-model tibial frame would require at least four rings each supported with three olive wires. An estimate based on the treatment of 40 patients was made. Single use of wires with reuse of other components (rings, rods nuts and bolts), either through recycling or sourced locally in Pakistan, was envisaged (Fig. 1a, b). As for locally sourced components, we established that roofing rods and nuts are the same size as the conventional Ilizarov equipment; these components are made of stainless steel [ST37-Din 1652 quality steel to mechanical property 4.6 grade to DIN.EN 20891-1, hot spelter galvanised to BS.1461-(1999) 45.5 HT and bright zinc plated to DIN.ISO.4042.AZK (BS.33202 Part 2)]. Rings, drills, hinges, wires, tensioners, washers, wire bolts and plates were donated/purchased from the manufacturer (Smith and Nephew Orthopaedics, Memphis, TN) and transported from UK by the team with significant costs reductions incurred. Surgical equipment processing and packaging was performed by the UK Hospital Trust Sterile Services Unit with sterile preparation and dressing packs donated by the Trust. In total, one tonne of equipment was collected, packaged and boxed with labels within 5 days (Fig. 1c).

Information sheets for patients were collected from our hospital but, despite being written in contained pictures on the post-operative management of Ilizarov fixators (e.g. pin-site care, post-operative exercises) and were to be given to each patient after surgery. Documents for the collating and collecting of operation records for each patient, including the surgical plan, were designed.

Special dispensations from the Pakistan Embassy in the UK provided emergency relief visas without costs for the surgeons and, following intervention by the Ambassador, Pakistan International Airlines allowed the equipment to be carried accompanied.

Following discussion with the local relief bodies, we were assigned to the Holy Family Hospital (a part of the Rawalpindi Medical College), a teaching hospital that normally caters for Obstetrics, Gynaecology and Ophthalmology. For the purposes of disaster relief efforts, orthopaedic procedures had commenced at the unit prior to our arrival with one local orthopaedic consultant supervising a team of others. The Limb Reconstruction Service provided by the team from the UK was to be in addition to the local services. Approval was obtained from the Principal of the Rawalpindi Medical College.

### The local team

Theatre staff supporting the UK team in Pakistan was comprised of local volunteers. Rapid training on the principles of Ilizarov fixator application techniques was undertaken by the senior member of the team in two seminar sessions. The theatres were equipped with general orthopaedic instruments with the visiting medical team providing the specialised Ilizarov instruments; sterilisation was carried out in an autoclave at the hospital (Fig. 1c). There were no image intensifier facilities in the operating theatres and no functional microbiology support.

There was support from local plastic and orthopaedic surgeons as well other medical and allied medical staff who were transferred from other peripheral hospitals. The local orthopaedic surgeons had a variable experience of the Ilizarov method and fixator. Nursing staff were trained in equipment use and post-operative care. Patients were taught pin-site care and encouraged to weight-bear and mobilise joints as part of the after-surgery protocol, thereby reducing the demand on the limited local physiotherapy services. Simple orthotics such as shoe raises was sourced from a local cobbler.

### Post-operative care

Analgesia was provided, but NSAIDs were avoided [9, 10]. Patients were instructed to bear weight through the limb when soft tissue conditions were deemed satisfactory by the local plastic surgeons, and usually after 5 days. Footwear with vulcanised rubber soles was provided and with heel raises as necessary. Check X-rays were obtained at the earliest opportunity.

Despite being a visit for a limited period and the nature of Ilizarov method treatments, there was confidence in the level of local expertise for post-operative care. The post-operative instructions included individualised treatment plans including gradual correction protocols, pin-site monitoring, radiological assessment and indicators for fixator removal.

The UK volunteer team made two visits. All patients treated in the first visit were reviewed by the team at the second visit. Patients who were operated on in the second visit were offered limb reconstruction only in collaboration with the local senior orthopaedic surgeon. Continued after-care of patients, although largely provided by the local orthopaedic surgeons, was carried out in collaboration with the UK team through electronic mail and telephone. A contrast to the first visit, where many cases were of fresh fractures, was the second visit where many patients had established, usually infected, non-unions. These patients had had external fixators and prior plastic surgical procedures.

## Results

### First visit

**Fig. 2:**
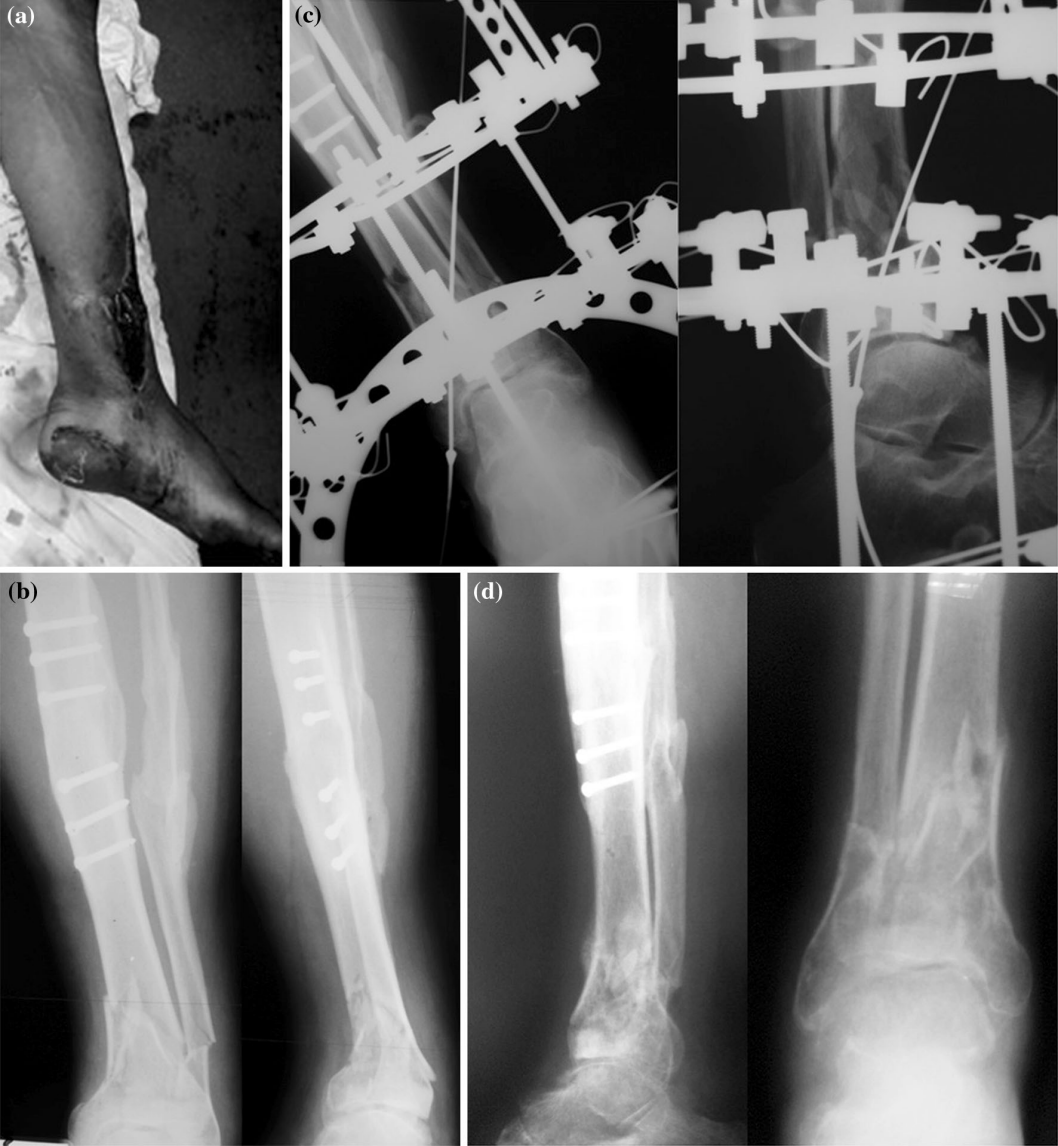
An example of a 56-year-old male with a 3B open Pilon type III fracture- treated with Ilizarov fixator. **a** Clinical picture, **b** preoperative X-rays, **c** X-rays 6 weeks post-operatively, **d** nine months post-operatively the fracture healed, and the frame was removed

This occurred 10 days after the earthquake of 8 October 2005 and lasted for a period of 5 days. All patients in the hospital had crush injuries as a direct consequence of the earthquake. Patients transferred to this unit were haemodynamically stable with single or multiple limb injuries. The patients selected for limb reconstruction were proposed by the local orthopaedic team as having injuries consistent with a likelihood of successful salvage. Eleven patients (age range 8–80 years) with 12 complex lower limb fractures (Table 1) were treated in 5 days. There were six male and five female patients; four were children (age range 8–15 years), and one adult female patient had bilateral pilon fractures. Nine cases were open Gustilo 3B fractures [3], and all fractures were associated with severe soft tissue injuries (Fig. 2). Three patients had other skeletal injuries. All patients were treated definitively with circular fixators (Table 2). Samples for microbiological assessment were not feasible due to the lack of facilities stated earlier. All patients received a cephalosporin combined with gentamycin post-operatively [4–6], which was obtained by donation from aid agencies. The antibiotics were stopped when soft tissue cover was carried out by the plastic surgeons.

**Table 1:** Demographic and injury description of patients treated during the first visit

Pat	Age/sex	AO [20]	Gustilo–Anderson	CL^a^	0P^b^	M/T^c^	N/V^d^
1	80/M	42-A2.3	Closed	IC2	–	–	–
2	15/F	42-A2.2	3B	–	IO4	MT3	NV1
3	50/M	43-A3.2, 43-C3.2	3B (L)	IC5 (R)	IO3	MT1	NV1
4	70/M	42-C2.3	3B	–	IO3	MT2	NV1
5	9/F	42-C2.2	3B	–	IO4	MT5	NV1
6	18/M	42-B2.3	3B	–	IO3	MT1	NV1
7	40/F	42-B2.2	3B	–	IO2	MT2	NV1
8	8/M	42-C3.3	3B	–	IO4	MT5	NV1
9	11/F	42-B3.3	3B	–	IO4	MT5	NV1
10	60/F	43-A3.3	Closed	IC2	–	–	–
11	24/M	42-C2.2	3B	–	IO4	MT3	NV1

Patient no. 3 has bilateral fractures

^a^AO classification for skin lesions in close fractures [21]

^b^AO classification for skin lesions in open fractures [21]

^c^AO classification for soft tissues [21]

^d^AO classification for neuro/vascular injury [21]

**Table 2:** Treatment that patients received during the first visit

Pat	Acute shortening (cm)	Treatment	Soft tissue procedure
1	–	3 rings IEF	–
2	3	5 rings IEF	SMF
3	2 (left)	5 rings IEF and corticotomy 4 rings IEF (right)	SMF (left)
4	3	4 rings IEF fibular tibialisation	SSG
5	6	3 rings IEF second-stage corticotomy for lengthening	SSG
6	2	4 rings IEF	GMF
7	5	4 rings IEF and corticotomy for lengthening	–
8	7	Fibular bone graft, 4 rings IEF	VD & FMF
9	5	4 rings IEF	VD & SSG
10	–	3 rings IEF	–
11	4	5 rings IEF	–

Patient no. 3 had bilateral fractures

*GMF* gastrocnemius vascularised pedicle muscle flap, *SSG* split skin graft, *SMF* soleus vascularised pedicle muscle flap, *VD* vacuum dressing, *FMF* free muscle flap as a second-stage procedure

The surgical objectives for these injuries were debridement, soft tissue cover and stable fixation to allow weight-bearing. Significant modifications of technique were necessary in the light of restricted facilities and prior staff experience. The procedures for adult patients were performed under regional anaesthesia and for children under general anaesthesia. Pre- and post-operative plastic surgical input was provided by local consultant plastic surgeons but with support from overseas plastic surgical trainees (Tables 2, 4).

The injured limb was washed with hydrogen peroxide and an iodine-based scrub solution and then washed again with normal saline. A formal prep was then carried out with an iodine-based solution. Soft tissue exploration and debridement was performed and the bone ends excised to bleeding margins using osteotomes. In the cases of non-unions where a previous flap was present, this was raised for access to the site.

The Ilizarov fixator was assembled over a peri-articular reference wire which was done by palpation of the transmalleolar axis or fibula head. This was helped by the use of a “sputnik” device—a long threaded rod connected at 90° by shorter threaded rods and placed on the tibial crest (Fig. 1d). The patients had very little body fat which facilitated the use of surface anatomy by palpation. Furthermore, the use of four rods in each ring segment allowed visual assessment within the tibia to ensure an orthogonal placement of the rings by eye. A construct consisting of two rings orthogonal to each segment of the tibia was done and the two constructs connected. In fractures involving the very distal part of the tibia, where a construct of two rings could not be made owing to the size of the segment, a half-ring with extension plates on each side was applied to the calcaneum as a foot extension to bridge the ankle joint (Tables 2, 4). In these cases, we opted not to use hinges (which would have allowed early ankle mobilisation) due to the lack of image intensifier facilities. A fibular osteotomy was performed as appropriate to aid acute shortening and compression of the non-union site and to stimulate limb blood flow [2]. In cases where acute shortening was not possible due to a large bony defect, a bone transport technique to fill the tibial defect was used [1, 7, 8].

Two patients required readjustment of the fixator after check X-rays were performed. All open wounds healed without clinical signs of infection. There were a number of pin-site infections treated with oral antibiotics; although no formal record of the number of pin-site infections was made, no patient required an exchange of wire due to infection. Fixators applied during the first visit were removed by the UK team during the second visit. Most patients accepted the leg length inequality that followed after resection.

### Second visit

The second visit, based at the same Holy Family Hospital, took place 3 months after the earthquake and was for 11 days. An image intensifier was available but shared with other routine lists. All the equipment taken in the first visit was available, and patients treated on the first visit were reviewed.

Three patients treated on the first visit elected to have leg lengths equalised; following discussion with the local orthopaedic team, these patients had a metaphyseal corticotomy performed for lengthening. In addition, thirteen new patients with complex non-unions, selected by the local orthopaedic surgeons or transferred from other hospitals, were treated (Table 3). There were ten females and three males (age range 18–55 years). Seven of the thirteen non-unions were clinically infected (there was no microbiology support available). All but two patients had previous treatment with monolateral fixators (AO type) and soft tissue procedures to cover exposed bone. One patient had had a circular fixator (Ilizarov) applied by another team, and one patient had been treated with traction for an extra-articular complex metaphyseal fracture of the distal tibia (43-A33).

**Table 3:** Demographic and injury description of patients treated during the second visit

Pat	Age/gender	AO [20]	Gustilo–Anderson	Initial treatment	Non-union	Infection
1	55/M	42-B23	3b	MXFIX/SMF	Y	Y
2	18/F	42-B33	3b	MXFIX/SSG	Y	Y
3	30/F	42-A11	3b	MXFIX/SMF	Y	Y
4	35/F	43-A22	3b	Ilizarov/SSG	Y	N
5	23/M	42-A22	3b	MXFIX/SSG	Y	N
6	41/F	42-B33	3b	MXFIX/SSG	Y	Y
7	23/F	42-A33	3b	MXFIX/SSG	Y	N
8	36/F	42-C22	3b	MXFIX/VAC/SSG	Y	N
9	22F	42-A33	3b	MXFIX/GMF	Y	Y
10	25/F	42-B22	3b	MXFIX/plaster	Y	Y
11	18/M	42-A23	3b	MXFIX/SSG	Y	Y
12	35/F	43-A33	Close	Traction	Y	N
13	25/F	42-C22	3b	MXFIX/SMF/SSG	Y	N

MXFIX, monolateral external fixator; initial treatment, treatment received by the patients before to be seen by the UK team

All patients underwent revision surgery using circular fixators (Table 4). The infected non-unions were treated surgically; flaps were raised, debridement carried out and stable fixation applied. All patients had a post-operative courses of a cephalosporin combined with gentamicin [4–6]. The duration of antibiotic treatment was decided by the local team.

**Table 4:** Treatment that patients received during the second visit

Pat	Acute shortening (cm)	Operation
1	2	3 rings IEF
2	14	Excision of more than 50 % of tibia/fibula, 4 rings IEF for bone transport
3	4	3 rings IEF
4	4	Readjustment of IEF, compression
5	–	4 rings IEF
6	6	4 rings IEF
7	–	4 rings IEF, compression
8	–	4 rings IEF
9	2	3 rings IEF
10	2	4 rings IEF in compression
11	5	4 rings IEF
12	–	3 rings IEF foot extension
13	–	4 rings IEF, compression

One patient who presented with an infected non-union and underwent excision of 14 cm of her tibia required a below knee amputation due to recurrent infection with purulent wound discharge. This was performed by the local team 2 months after the Ilizarov frame application.

The remaining cases united without further events.

## Discussion

The use of Ilizarov fixator is well established for limb salvage and complex fractures with severe soft tissue injuries [7, 11–14]. These principles were used for a mass casualty setting in a disaster zone (Fig. 2). These included adequate wound debridement, stabilization with plastic surgical coverage and early mobilisation. Despite difficulties that exist in such circumstances, this experience suggests that the application of basic Ilizarov principles in a disaster zone can be as effective as in established limb reconstruction and trauma units.

A major factor contributing to the overall success was the detailed pre-visit planning and effective collaborative utilisation of local personnel and facilities. This enabled the UK team to manage patients without significant compromise to the surgical objectives despite a lack of trained staff and facilities.

Owing to a lack of microbiological support, wound cultures from debridement were not obtained. There is some evidence to suggest such a practice may not be necessary; Lee et al. [15] and Valenziano et al. [16] have questioned the use of wound cultures, and a study by Okike et al. [17] did not recommend the routine use of cultures before and after debridement [17].

In dealing with supply and cost of medical equipment within a limited budget (from donations), we opted for cheaper alternatives that would not compromise patient care. A similar practice has been reported for developing countries [18]. None of these instruments failed, and this approach enabled the spare money to purchase a larger number of specialised equipment (rings, wires, wire bolts, hinges, etc.). In the setting of a major disaster scenario, we consider this to be an acceptable compromise in order to treat more patients.

Amputation was not considered a first-line treatment for this group of patients because of the poor availability of prosthetic limb centres in Pakistan and the questionable long-term cost of limb reconstruction versus prosthetics for young patients. The majority of these patients were young and were not from urban centres but evacuated from rural Kashmir which, following the earthquake, had limited infrastructure remaining. The cost of prosthetics over a young patient’s lifetime, which in most cases would be self-funded, would be very high. McKenzie et al. [19] suggested reconstruction for the treatment of injuries below the distal part of the femur typically resulted in functional outcomes equivalent to those of amputation. This conclusion would not be applicable in a setting with inadequate provision of prosthetics.

The limb reconstruction cornerstone techniques of debridement, stable fixation and functional have a heightened significance for limb injuries in a mass casualty situation. Patients who had good debridement, stable fixation and were walking early did better and avoided complications that required more advanced techniques of limb reconstruction on the second visit.

### Recommendations

1. Plan effectively
  Local resources will be stretched or unavailable; therefore, ensure that you have all equipment that you may possibly need.
  The ideal team should have experienced anaesthetists, orthopaedic surgeons and plastic surgeons.
  Accept the expertise of the local surgeons and care givers.
  Keep to simple proven techniques.
2. Learn about your patients
  Adult patients required 140-mm-diameter rings and even 120 mm was found to be adequate. Rings of 180 mm diameter were unnecessary.
  Prepare information sheets appropriate to the country; use illustrations liberally to overcome issues of illiteracy.
  Do not underestimate the patient.

## Summary

With appropriate organisation and the application of basic Ilizarov principles, successful limb salvage was possible for a small group of patients in the absence of specialised facilities and personnel.
